# Circadian rhythm melatonin in acute and stabilized schizophrenic patients

**DOI:** 10.1192/j.eurpsy.2025.2214

**Published:** 2025-08-26

**Authors:** E. Diaz Mesa, A. Morera-Fumero, I. Zebenzui-Moreno Gonzalez, R. de Leon Hernandez, P. Abreu-Gonzalez, M. D. R. Cejas-Mendez, L. Fernandez-Lopez

**Affiliations:** 1 Psychiatry, University Hospital of the Canaries; 2 Department of Internal Medicine Dermatology and Psychiatry, School of Medicine; 3 Physiology, University of La Laguna, La Laguna, Spain

## Abstract

**Introduction:**

Melatonin is a neurohormone plays a role in the development and course of schizophrenia.Melatonin also has an important role in regulating circadian rhythms, so melatonin secretions have been suggested as markers for circadian dysfunctions in schizophrenic patients. Some studies have assessed the circadian profiles of melatonin secretion in schizophrenic patients, showing that the nocturnal circadian rhythm of plasma melatonin secretion was disrupted in medicated chronic schizophrenics (Jiang H. K. & Wang J. Y. Diurnal melatonin and cortisol secretion profiles in medicated schizophrenic patients. *Journal of the Formosan Medical Association. Taiwan yi zhi 1998 97*(12), 830–837). Nocturnal plasma melatonin secretion was also reduced in the drug-free schizophrenic patients (Monteleone P. et al. Depressed nocturnal plasma melatonin levels in drug-free paranoid schizophrenics. *Schizophrenia research* 1992;*7*(1), 77–84). These findings could suggest the presence of abnormal melatonin rhythm in schizophrenics, which may be related to the pathophysiologic process itself. However, it is not known whether there are melatonin secretion significant differences in acute phase and clinical stabilization phase of schizophrenic patients.

**Objectives:**

The aim of this study is to assess the melatonin secretion profile in acute phase and clinical stabilization phase of schizophrenic patients to know if exists circadian rhythm and to compare both measures.

**Methods:**

Our sample consists in 48 patients that have been study in two clinic phases: acute phase and after discharge,clinically stabilized. Blood samples were collected in the morning (12:00 hours) and at night (00:00 hours) by venipuncture in reclined position with mask that covers the eyes. Serum levels of Melatonin were measured in acute phase and stabilized phase, after discharge. Melatonin was measured by means of ELISA (Enzyme-linked immunosorbent assay) techniques. The relationship between quantitative variables at two times of the same marker were analyzed by T-Student test.

**Results:**

All comparisons reach a degree of statistical significance, which we can interpret as meaning that there is a circadian rhythm at the two moments measured. Furthermore there significant difference between acute phase of the schizophrenia respect control phase, three months after discharge.

**Conclusions:**

In this study we can hypothesize that Melatonin has circadian rhythm when the patients are in acute psychosis and when are stabilized. MLT levels increase at control, a fact that we can interpret as there is an increase in antioxidants levels in the clinical stabilization phase, this increase being even greater at the three-month control, which may be attributable to the treatment due to its antioxidant properties or to the disease itself. The results of this biomarker could be interpreted as is useful variable for the prognosis.

**Disclosure of Interest:**

None Declared

